# Interleukin-33 in Malignancies: Friends or Foes?

**DOI:** 10.3389/fimmu.2018.03051

**Published:** 2018-12-20

**Authors:** Jia-Xin Shen, Jing Liu, Guo-Jun Zhang

**Affiliations:** ^1^Chang Jiang Scholar's Laboratory, Shantou University Medical College, Shantou, China; ^2^Department of Hematology, The First Affiliated Hospital of Shantou University Medical College, Shantou, China; ^3^Department of Physiology, Shantou University Medical College, Shantou, China; ^4^The Cancer Center and the Department of Breast-Thyroid Surgery, Xiang'an Hospital of Xiamen University, Xiamen, China

**Keywords:** IL-33, cytokine, cancer, immunology, therapy

## Abstract

The human Interleukin-33 (IL-33), a member of the IL-1 family, is the cytokine as a cell endogenous alarmin, released by damaged or necrotic barrier cells (endothelial and epithelial cells). The signal transduction of IL-33 relies on recognition and interaction with specific receptor ST2, mainly expressed in immune cells. In both innate and adoptive immunity, IL-33 regulates the homeostasis in response to stress from within/out the microenvironment. Various, even negative biofunctions of IL-33 pathways have now been widely verified in pathogenesis among immunological mechanisms, like Th2-related immune-stimuli, inflammation/infection-induced tissue protectors. A larger versatility in studies of IL-33 on malignancies now focuses on: (1) promoting myeloid-derived suppressor cells (MDSC), (2) intervention toward CD8^+^ T, Natural Killer (NK) cell infiltration, group 2 innate lymphoid cells (ILC2) proliferation, dendritic cells (DC) activation, and (3) inhibiting tumor growth and/or further metastasis as an immunoadjuvant. Although IL-33 functioned pro-tumorigenically in various cancers, for some cancer types the findings so far are controversial. This review begins from a summarized introduction of IL-33, to its remarkable implications and molecular transduction pathway in malignant neoplasms, ends with latest inspiration for IL-33 in treatment.

## Introduction

**Figure 1:**
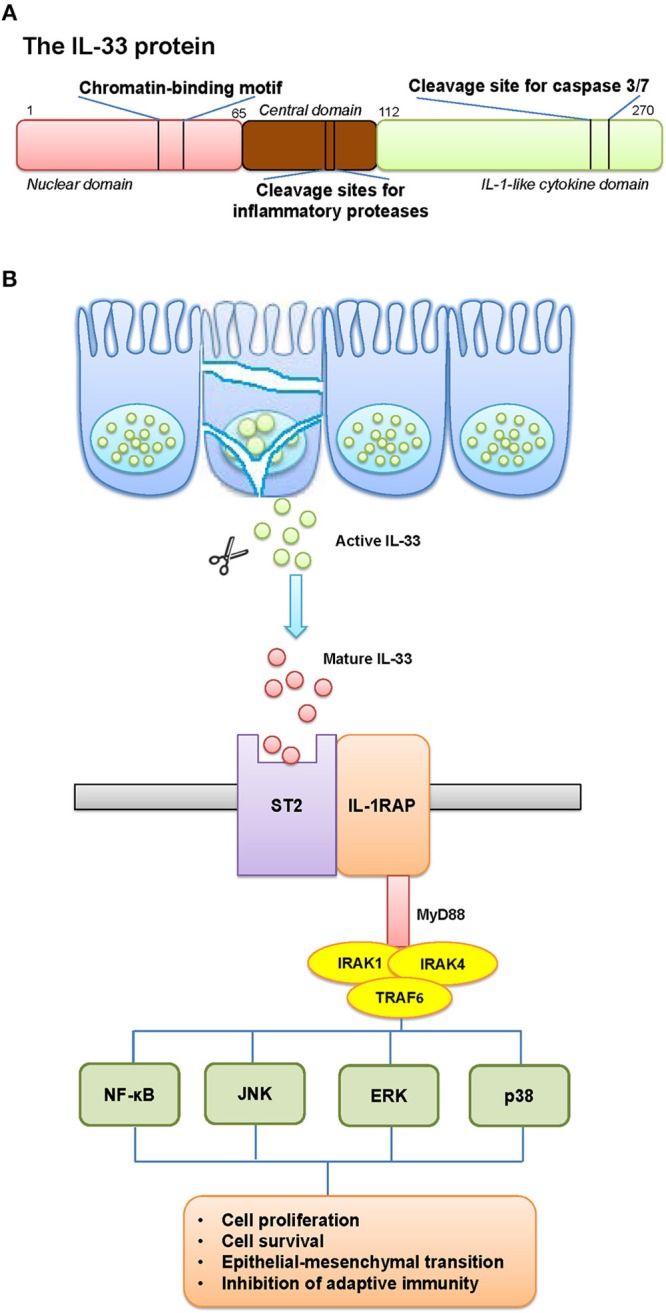
The schematic elucidation of IL-33. **(A)** The schematic structure of IL-33 protein and its critical domains. **(B)** The pathway involved IL-33 and its receptor. **(A)** IL-33 gene is constitutively located on the short arm of chromosome 9 at 9p24.1. The structure of IL-33 protein contains two evolutionary conserved domains: a chromatin-binding motif, and a cleavage site for inflammatory proteases and apoptotic caspases. **(B)** The release mechanisms of IL-33 can occur by mechanical and oxidative stress, necrotic cell death, or cell activation that functions as an alarmin. IL-33 is rapidly released from cells during necrosis or tissue injury, and signals through a cell surface receptor complex, ST2 (IL-1 receptor-like 1, IL1RL1) and IL1RAcP (IL-1 receptor accessory protein), to initiate inflammatory pathways in immune cells. This clustered domain recruits signaling adaptor and kinases (including MyD88, IRAK1/4, TRAF6) in order to activate the transcription factors in tumor cells. The possible results would be a generated cancer-related inflammatory microenvironment, with tumor-promoting effects.

Cytokines are central mediators between cells in the inflammatory tumor microenvironment, in which Interleukin-33 (IL-33) is considered as an alarmin released after cellular damage. IL-33 was discovered as a member of the IL-1 family of cytokines. The IL-1 gene family contains 11 members (IL-1α, IL-1β, IL-1RA, IL-18, IL-36RA, IL-36α, IL37, IL-36β, IL-36γ, IL-38, IL-33), which induces a complex network of pro-inflammatory cytokines, and regulates and initiates inflammatory responses, via expressing integrins on leukocytes and endothelial cells (1). IL-33 gene is constitutively located on the short arm of chromosome 9 at 9p24.1. Sequencing alignment toward human and murine IL-33 revealed two evolutionary conserved domains; a chromatin-binding motif, and a cleavage site for inflammatory proteases and apoptotic caspases (Figure 1A). The discovery of two ST2-binding sites further confirmed an exerted binding of IL-33 to a heterodimer, formed by the specific primary receptor ST2 and the IL-1 receptor accessory protein (2, 3).

The full-length IL-33 contains 270 amino acids in human and 266 in mice, which harbors a homeodomain-like helix-turn-helix domain presumably allowing to bind to DNA (4). The release of IL-33 can be associated with mechanical and oxidative stress, necrotic cell death, or cell activation through ATP signaling in the absence of cell death (5). IL-33 is rapidly released from cells during necrosis or tissue injury, and signals through a cell surface receptor complex, ST2 (IL-1 receptor-like 1, IL1RL1) and IL1RAcP (IL-1 receptor accessory protein), to initiate inflammatory pathways in immune cells, such as type-2 innate lymphoid cells (ILC2), mast cells and natural killer (NK) cells (6). Although advances have been made, mechanisms regulating the alarmin activity of IL-33 remain poorly understood (Figure 1B).

Human nervous tissues, barrier structures with widespread of endothelial, epithelial and fibroblast-like cells that are exposed to the environment, were indicated high level of constitutive expression of IL-33. However, inducible IL-33 by inflammation catches more attention of researchers from classical cases with chronic obstructive pulmonary disease, inflammatory responses to suffered graft-vs.-host diseases after bone marrow transplantation. Most recently, tissue fibrosis, mucosal healing, and wound repairmen were as well found to be possible initiatives of IL-33 during inflammation. There are outcomes of cross-talks and interactions within DNA, mRNA, and protein levels. Strict regulation starts from nuclear localization and chromosome association of IL-33, where nuclear IL-33 functions as a transcriptional repressor when overexpressed in chronically in?amed tissues from patients with rheumatoid arthritis and Crohn's disease (7, 8).

### The Roles of IL-33 in Malignancies

**Figure 2:**
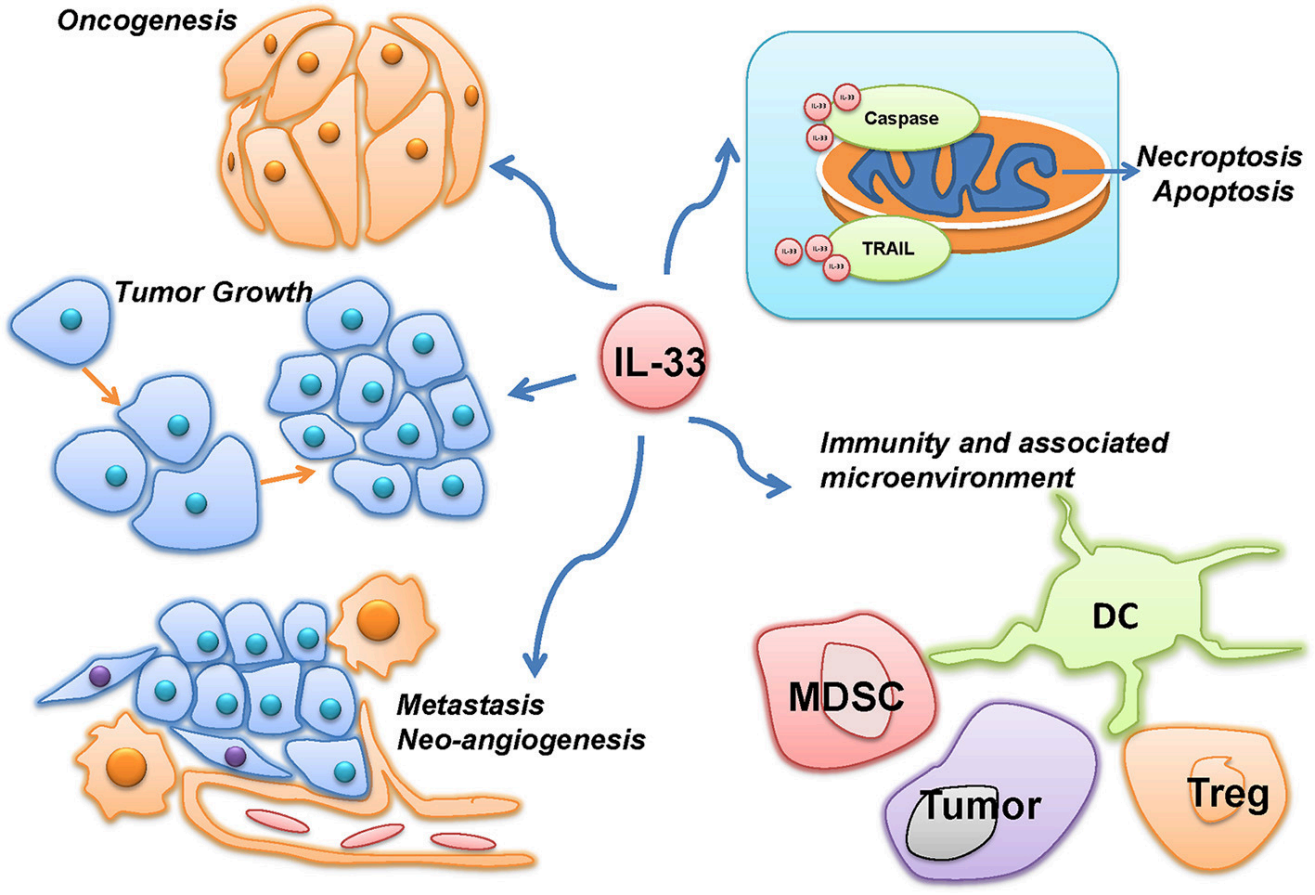
The multiple roles of IL-33 in malignancies. IL-33 was reported to be involved in the different hallmarks of cancer, such as oncogenesis, tumor growth, metastasis, neo-angiogenesis, and evading programmed cell death. However, as an immune-associated factor, IL-33 was also demonstrated to affect the immunity and associated microenvironment of cancer through immune cells, such as myeloid-derived suppressor cells (MDSC), Dendritic cells (DC), and Regulatory T cells (Treg).

The multiple roles of IL-33 in malignancies were summarized in Figure 2. Most of the current studies on IL-33's multiple roles in cancers focus on tumor microenvironment, tumorigenesis and tumor-associated inflammatory responses. In head-and-neck-squamous cancers, cancer associated fibroblasts was found releasing IL-33, sequentially leading migration and invasion through epithelial-to-mesenchymal transition (9). Data from tongue cancer patients witnesses a worse prognosis with higher level of IL-33 or ST2. Well-designed tissue comparison assays showed an elevated IL-33 and IL1RL1 or ST2 in tissue of both human breast cancer and non-small-cell-lung cancer (NSCLC), compared to adjacent non-tumor tissues. Similarly, high level of serum IL-33 also indicated a poor prognosis in patients with these two types of cancers (10–13). Related mechanism includes genomic instability and mutation, epigenetic modification, apoptosis resistance to cancer-initiated cells and increases of cancer metastasis.

### Immunity and Associated Microenvironment

The process of tumor development can trigger anti-tumor immune responses. The type 1 immune response is a critical component of cell-mediated immunity, which includes tumor-induced IFN-γ-producing Th1 cells, cytotoxic T lymphocytes, NK T cells, and γδ T cells, to limit tumor growth and metastasis (14). Since inflammation is another important component in malignancies, it drove more studies on how IL-33 plays roles in improving cancerous surveillance and immunity against tumor. Gao et al. successfully gained the increased innate immunity of CD8(+) and NK cells by overexpressing IL-33 in tumor-bearing mice. The metastatic potential in models of B16 melanoma and Lewis lung carcinoma were significantly attenuated by transgenic expression of IL-33 (15). When depleting CD8(+) T cells and NK cells, pulmonary metastasis was significantly increased, indicating that IL-33 can mediate the anti-tumor immunity of CD8(+) T cells and NK cells.

However, other researches considered IL-33 promoted cancer progression through diminishing innate anti-tumor immunity and increasing intra-tumor accumulation of immunosuppressive cells in transgenic mice with breast cancer. Jovanovic et al. detected a time-dependent increase of endogenous IL-33 at both mRNA and protein levels in 4T1 breast cancer model and pulmonary metastasis during cancer progressions. In IL-33-treated mice, intra-tumor NKp46(+) NKG2D(+) and NKp46(+) FasL(+) cells were markedly reduced, while PBS-treated ST2-deficient mice had increased frequencies of these tumoricidal NK cells compared to that in untreated wild-type mice (16). Similarly, IL-33 increased IFN-γ by CD8(+) T and NK cells in tumor tissues, thereby inducing the microenvironment accessible to tumor eradication (17).

The tumor microenvironment, in which the tumor exists, constantly interacts with tumor cells. The developing tumor itself can promote anti-tumor immune responses by recruiting cytotoxic T lymphocytes, T helper 1 cells, and NK cells; so as to influence the microenvironment by releasing extracellular signals, promoting tumor angiogenesis and/or peripheral immune tolerance (14, 18). At the same time, the immune cells involved can affect the growth and evolution of tumor cells (19). Immune suppression specific to tumor and inflammatory stimuli may therefore display a cancer-initiated action, where cytokines are the central mediators (20, 21). IL-33 was proved to be a robust inducer of T helper 2 cells (releasing cytokines like IL-4/5/6/10/13) in tumor microenvironment, but a suppressor to the secretion of T helper 1 cells (cytokines like IL-12, IFN-γ) (22). Recent data revealed the role of IL-33 in several cancers, indicating dual functions as a damage-associated molecular pattern, or nuclear factors mediating gene expression (23).

#### Oncogenesis

Environmental factors (nicotiana or alcoholic use), and infections (hepatitis B virus), were indicated as triggering factors for minor changes in lungs, heart and livers (24). These were later proved to initiate a higher rate of neoplastic growth, followed by chronic inflammation to targeted organs and/or tissues. Therefore, the tumor-associated inflammatory pathways to cancer development are research hotspots.

Tumors often locally accumulated Treg cells, preventing tumor clearance. In intestinal tumors from APC^Min/+^ mice, researchers found that a high level of cytokine IL-33 were preferentially expanded in KLRG1^+^ GATA3^+^ Treg cells, sequentially to activate E-cadherin ablation and increase β-catenin signals in epithelial cells (25, 26). In another remarkable study of patients with metastatic colon cancer, higher expression of IL-33 in cancer tissues was significantly associated with poorer survival. Similarly, the study pointed out that IL-33 activated core stem cell genes via ST2 signaling pathway, recruited macrophages and stimulated stem-like characters to promote carcinogenesis of colon cancer cells (27, 28). Another new function of IL-33 discovered from non-basophilic leukemia, a rare subtype of acute myeloblastic leukemia, indicated IL-33 enhanced the basophilic differentiation of MYB-GATA1 expression, demonstrating a new role of IL-33 in leukemic cells and CD34-positive primary cells (29).

#### Tumor Growth

IL-33 is a cytokine implicated in mutual modulation of not only anti-tumor immunity but tumor growth. Inflammatory responses can be triggered by tumor growth through releasing danger signals and expression of tumor antigens (14). Nevertheless, tumors progress by enlisting and driving the dominance of immune suppressive cell types such as Treg and myeloid-derived suppressor cells, as well as myeloid cells that produce cancer-promoting factors (18, 30, 31). Moreover, IL-33 also promoted the growth and metastasis of solid cancers, such as gastric cancer, colorectal cancer, ovarian cancer, and breast cancer (32, 33). Wang et al. reported blocking IL-33 activities restricted tumor growth of NSCLC xenografts, indicating IL-33 blockade as a novel therapeutics for NSCLC patients (34).

Conversely, other studies pointed out anti-cancer activities of IL-33 to inhibit tumor progression in cellular levels and animal models. Qin et al. showed that recombinant IL-33 dramatically repressed the leukemia growth and prolonged the survival of leukemia-bearing mice by increasing IFN-γ production of leukemia-reactive CD8+ T cells (35). Tumor expression of IL-33 was also reported to inhibit tumor growth and favor tumor eradication by modifying the tumor microenvironment through CD8^+^ T cells (17). Put together, it is proposed that IL-33 might exert the anti-cancer activities of suppressing tumor growth under certain circumstances.

#### Metastasis and Neo-Angiogenesis

Cancer metastasis is one of the severe outcomes in most patients with malignancies. It is characterized by rapid and uncontrolled proliferation with high ATP demands, which requires a high rate of glucose uptake. In the surface of NSCLC cells, IL-33/ST2 pathway upregulated membrane glucose transporter 1 to enhance their glucose uptake and glycolysis to meet the ATP switch of metastasis (36).

Saranchova et al. described a new mechanism of immune-surveillance in cancer. They found metastatic carcinomas expressed low levels IL-33 and antigen processing machinery (APM), compared to syngeneic primary tumors. Supplementation of IL-33 in metastatic tumors restrained tumor growth rates and frequencies of circulating tumor cells by upregulating APM and functionality of major histocompatibility complex (MHC)-molecules, indicating the inhibition function of IL-33 to primary tumors in cancer immune-surveillance and losing that function during metastatic transition as immune escape (37). Evidences pointed out IL-33 as a possible inducer and prognostic marker of cancer metastasis by way of mechanisms like immune regulation, with intensive cytotoxic activities of NK cells and increased systemic Th1 and Th17 cytokines (38).

Other malignancies witness proof of IL-33 to metastatic process. The soluble form of the IL-33 receptor (sST2), resisting IL-33-induced angiogenesis, is downregulated in metastatic cells compared with low-metastatic colorectal cancer cells (39). Wang et al. determined that IL-33 overexpression enhances robust outgrowth and metastasis *in vitro*/*in vivo*, while genetic knockdown of IL-33 limited the progression of NSCLC (40). In lines with these findings, epithelial ovarian cancer knocked down of IL-33 gene had reduced metastatic potential, while ectopic IL-33 promoted the migratory and invasive capacity. Underlying mechanisms clarified sST2 might block the ERK and JNK signaling pathways, where IL-33 in return, was regarded as a prognosis markers and targets for ovarian cancers (41). In gastric cancer cells, IL-33 promoted cancer migration and invasion through stimulating the secretion of MMP-3 and IL-6 via aberrant activation of ST2-ERK1/2 pathway (42). Taken together, IL-33 may promote cancer development and metastasis through different pathways.

#### Cancer Cell Death

Different modes of cell death regulate inflammation by modulating professional phagocyte activation (43). The topic concerning IL-33 initiated cancer cell death is still emerging. It is likely to influence a range of diseases, but evidence for IL-33 limited to only a few examples.

Apoptosis is one of the classically understood processes of cell death that denotes a specific caspase-8–dependent programmed cellular death (44). Ye et al. released a recent study that IL-33 prevent cancer cells against platinum-induced apoptosis via the JNK pathway in gastric cancer cells (45). Similarly, in colon cancer cells, IL-33 stimulated cell sphere formation and prevented chemotherapy-induced tumor apoptosis (28). One exception was noticed in MIA PaCa-2, a pancreatic cancer cell line, that not only colonies and proliferations rate, but relative caspase-3 activities were attenuated in the presence of IL-33. Subsequent anti-proliferative effect on cancer cells even correlated with down-regulated anti-apoptotic molecule FLIP and up-regulated pro-apoptotic molecule TRAIL (46). Since unidentified mechanisms of IL-33 in basic research, we need further experiments to clarify the possibly opposite effects in different situations, like inflammatory stages, cell types or even the concentration of IL-33.

Based on signaling pathway studies, IL-33 may also be involved in co-activation of receptor-interacting protein 1/3 (RIP1/3) kinases, subsequently to damage, press and chronic inflammation (47, 48). This discovery shed light on another versatility of IL-33, an atypical cell death named necroptosis. Unlike apoptosis, this caspase-8-independence cell death allows the cell to bypass caspase activation (49, 50). In liver malignancies, inflammatory stimuli are predominantly driven to tissue-resident Kupffer cell from by bacterial and viral infections. Necroptosis of Kupffer cells released the alarmin IL-33 to trigger basophil IL-4, which successively recruited macrophages proliferation. This ultimately activated the macrophages and thereby achieved to liver homeostasis (51). While in cervical cancer, IL-33 related necroptosis could work as a possible pioneer for immunotherapy in cervical cancer. This HPV-induced cancer was found a rather low level of cytokine, indicating a stimulated releasing of IL-1α for an induced necroptotic cell death (52). Still, potential mechanisms on these studies need further clarified.

### Therapeutic Strategies for IL-33 in Cancers

Understanding of direct and indirect effects of IL-33 would be important for profound therapeutic implications, especially in the realm of cancer immunotherapy. By using what scientists learn about the immune system, several synthetic molecules are created to attack a tumor more precisely and effectively (53). Current cancer immunotherapies include cytokines, monoclonal antibodies, and lymphocytes that will enhance existing antitumor immune responses (54). Cytokine-based immunotherapy has been extensively investigated in the treatment of malignancies. These immune-modulating effects allow interleukin-related treatment to provoke an immune response to chronic rheumatic diseases and attenuate disease progression of pathogenic conditions. However, only a few agents, such as interferon and IL-2, have proven to have sufficient clinical benefits to justify their more widespread use (55). Many preclinical studies demonstrated the antitumor effects of Th1 cytokines, to which IL-33 belongs, while clinical efficacy still limited.

Since IL-33 dually drives the immune system in either responsiveness/activation or tolerance/inhibition, IL-33 administration to the status of immune system dynamics controversially direct immune response and determine outcome (56). Any underlying natural bimodal homeostatic dynamic will be critical principal determinant of clinical efficacy. It is suspected that cancer therapy will be to analyze accurately the patient's underlying tumor immune response in a serial manner, then appropriately and accurately synchronize therapy with immune fluctuation (57). This time-dependent dynamic aspect of the immune response in the cancer patient has been largely overlooked in the past and has prevented us from being able to observe how our *in vivo* therapeutic approaches are influencing the immune response to produce the observed clinical effects.

IL-33 has been possibly considered as an immune adjuvant for vaccine therapy. Indeed, IL-33 functions as a promoter to memory T cell immunity in transgenic melanoma mice, thereby mediating a microenvironment that favors tumor rejection. Based on insights from Villarreal, this immunoadjuvant effects in an HPV-associated model is critical for protective immunity (58). Besides, increased IL-33 in pathological settings including tumor immunotherapy, viral infection and graft-vs.-host diseases, suggest that IL-33 overexpression might elicit potent antitumor immunity (59). Thus far, mouse models provide most of the information at hand. To what degree these observations are applicable to real patients is still unclear. Experimental evidences are currently lacking that clearly indicate the suitability of such an approach.

As a tumor biomarker, IL-33 could be versatile serving as a therapeutic target in patients. In breast cancers, estrogen receptor expressions account for most clinical cases. Thanks to hormone therapy with tamoxifen, prognoses of ER-positive breast cancer are significantly improved. While on the other hand, endocrine resistance to tamoxifen led to commonly-found compromised efficacy of hormone therapy where IL-33 played crucially. Knockdown of IL-33 is likely to correct this problem and resistance to tamoxifen-induced tumor growth inhibition could therefore be reversed (60). Similar cases derived from previous studies in lung and gastric cancer, in metastatic prostate carcinomas, and glioblastoma (61). These findings point out new connection betweenIL-33 and cancer pathogenesis and pinpoint IL-33 promising to optimize therapy in clinical practice.

### Future Perspectives

The role of IL-33 in inflammatory diseases has been widely discussed since decades ago (62). This review highlights the remarkable span and diversity of its modulatory potency in tumors. Interestingly, our perspectives in IL-33 has now extended beyond its previous identification as an inducer of immune responses to that of a potency in chronic inflammation and timely activation by malignancies. However, many questions remain unraveled, such as the regulatory elements, the bioactive forms, and the inner homeostasis of IL-33. A study of network map insights on IL-33-mediated crosstalk in the pathogenesis of acute and chronic inflammatory diseases (63). It revealed various roles of classical signaling modules like ERK1/2, NK-κB, and PI3K/AKT, etc. that played in disease development, which in no doubt left us with new inspirations to tumor-related development. In therapeutic practices, IL-33-targeted antibody has only been put into clinical trials for asthma. Other approaches were newly recommended, but cautions should be exercised due to the many immune responses and the potential for driving cancer development.

## Author Contributions

JL and G-JZ contributed conception and design of the study. JL and J-XS organized the database, searched literatures, structured, and drafted the manuscript carefully. JL and G-JZ revised the original manuscript critically. All authors contributed to manuscript revision, read, and approved the submitted version.

### Conflict of Interest Statement

The authors declare that the research was conducted in the absence of any commercial or financial relationships that could be construed as a potential conflict of interest.
